# An *in vitro* model to study suction events by a ventricular assist device: validation with clinical data

**DOI:** 10.3389/fphys.2023.1155032

**Published:** 2023-07-25

**Authors:** Maria Rocchi, Christoph Gross, Francesco Moscato, Thomas Schlöglhofer, Bart Meyns, Libera Fresiello

**Affiliations:** ^1^ Unit of Cardiac Surgery, Department of Cardiovascular Sciences, Katholieke Universiteit Leuven, Leuven, Belgium; ^2^ Department of Cardiac Surgery, Medical University of Vienna, Vienna, Austria; ^3^ Center for Medical Physics and Biomedical Engineering, Medical University of Vienna, Vienna, Austria; ^4^ Ludwig Boltzmann Institute for Cardiovascular Research, Vienna, Austria; ^5^ Austrian Cluster for Tissue Regeneration, Vienna, Austria; ^6^ Department of Cardiac Surgery, University Hospitals Leuven, Leuven, Belgium; ^7^ Cardiovascular and Respiratory Physiology, University of Twente, Enschede, Netherlands

**Keywords:** ventricular assist device, suction, cardiovascular simulator, hybrid simulator, physiological controllers, validation

## Abstract

**Introduction:** Ventricular assist devices (LVADs) are a valuable therapy for end-stage heart failure patients. However, some adverse events still persist, such as suction that can trigger thrombus formation and cardiac rhythm disorders. The aim of this study is to validate a suction module (SM) as a test bench for LVAD suction detection and speed control algorithms.

**Methods:** The SM consists of a latex tube, mimicking the ventricular apex, connected to a LVAD. The SM was implemented into a hybrid *in vitro*-*in silico* cardiovascular simulator. Suction was induced simulating hypovolemia in a profile of a dilated cardiomyopathy and of a restrictive cardiomyopathy for pump speeds ranging between 2,500 and 3,200 rpm. Clinical data collected in 38 LVAD patients were used for the validation. Clinical and simulated LVAD flow waveforms were visually compared. For a more quantitative validation, a binary classifier was used to classify simulated suction and non-suction beats. The obtained classification was then compared to that generated by the simulator to evaluate the specificity and sensitivity of the simulator. Finally, a statistical analysis was run on specific suction features (e.g., minimum impeller speed pulsatility, minimum slope of the estimated flow, and timing of the maximum slope of the estimated flow).

**Results:** The simulator could reproduce most of the pump waveforms observed *in vivo*. The simulator showed a sensitivity and specificity and of 90.0% and 97.5%, respectively. Simulated suction features were in the interquartile range of clinical ones.

**Conclusions:** The SM can be used to investigate suction in different pathophysiological conditions and to support the development of LVAD physiological controllers.

## 1 Introduction

Continuous Left Ventricular Assist Devices (LVADs) are blood pumps that operate at a constant speed and propel blood from the left ventricle to the aorta to support the failing ventricle. In the last decade, LVAD technology has evolved and improved dramatically, from second generation of LVADs, typically axial flow pumps with mechanical bearings to keep the rotor in place, to third generation LVADs, centrifugal flow pumps with hydrodynamic and magnetic bearings (Goodman et al., 2022). As a result of the late improvements in terms of technology miniaturization and durability, LVADs are largely used in chronic settings as destination therapies for advanced stage heart failure patients. (Teuteberg et al., 2020). This opens new challenges in terms of hemodynamic interaction between LVAD-patient in daily life. (Fresiello et al., 2017; Gross et al., 2018). Indeed, a main limitation of such devices is the lack of sensitivity to the preload of the supported ventricle. (Fukamachi et al., 2013). Given the changing perfusion demand of a patient during the day, hemodynamic changes may lead either to an underpumping of the ventricle, causing pulmonary congestion, (Gross et al., 2018), or to an overpumping of the ventricle, causing the emptying of the ventricle and consequent collapse of the ventricular walls, thus a suction event (Reesink et al., 2007; Gross et al., 2020). Suction events should be avoided as they can cause damage both to the patient, exacerbating the heart failure symptoms, and to the medical device in terms of impeller instabilities and possible stops of the pump flow (Reesink et al., 2007; Salamonsen et al., 2015; Moss et al., 2017; Gross et al., 2020). Potential consequences are: damage to blood components and to ventricular walls with possible induction of ectopic rhythms, creation of hematoma or necrosis, and thrombus release (Reesink et al., 2007; Salamonsen et al., 2015; Sen et al., 2016; Moss et al., 2017; Aissaoui et al., 2018). Suction is a relatively common phenomenon among LVAD patients, Gross et al. (Gross et al., 2020) identified a range of 3%–61% of heartbeats per hour with suction in an observational study of 10 outpatients monitored continuously for 15 days.

Several research groups and companies are studying physiological pump controllers to change the LVAD speed to avoid suction. (Hatoh et al., 1999; Vollkron et al., 2004; Karantonis et al., 2006; Ferreira et al., 2007; Maw et al., 2021; Maw et al., 2022). Therefore, there is a need for an *in vitro* test bench with high fidelity and versatility to capture the wide range of suction characteristics observed in the clinics and capable of providing repeatable test scenarios for pump controllers. In the literature, different *in vitro* models have been developed to recreate suction, either with a pneumatically operated pinch valve or with a collapsible element. (Gwak et al., 2005; Ferreira et al., 2012; Ochsner et al., 2013; Stevens et al., 2014; Pauls et al., 2016a; Pauls et al., 2016b; Petrou et al., 2018a; Petrou et al., 2018b). In the first case, a valve is used to cut the flow off near the inflow cannula of the LVAD when the volume reached values below a threshold (Stevens et al., 2014). The main limitation of this approach is that the mechanics of the ventricular walls collapse could not be replicated, and only a full occlusion could be simulated. Another approach foresees the use of a collapsible element to either mimic the ventricular apex or the entire ventricular cavity (Gwak et al., 2005; Ferreira et al., 2012). This approach allows for a better representation of the phenomenon of suction, reproducing both a partial and full collapse of the ventricular walls. The main limitation of these studies is the lack of accuracy in replicating the physiologic status of the ventricle during suction.

Our research group has developed a suction module consisting of a compliant representation of the ventricular apex that overcomes the limitations of the aforementioned setups. Indeed, the suction module is implemented in a high fidelity simulator of the entire closed loop cardiovascular system. (Rocchi et al., 2021a; Rocchi et al., 2021b). The cardiovascular simulator can represent different (patho-)physiologies by tuning cardiac parameters (e.g., heart rate, left/right ventricular systolic and diastolic status), vascular parameters (e.g., circulating blood volume, systemic vascular resistance) and LVAD speed level. (Rocchi et al., 2021a). As such, the suction module has the ability of reproducing intermittent and continuous suction with different severity levels according to the underlying hemodynamic condition.

The aim of this study is to further validate this model against a database of LVAD waveforms (with and without suction) recorded in patients at the Medical University of Vienna (Gross et al., 2020; Maw et al., 2021; Moscato et al., 2021).

## 2 Methods and methods

### 2.1 *In silico-in vitro* setup

**FIGURE 1:**
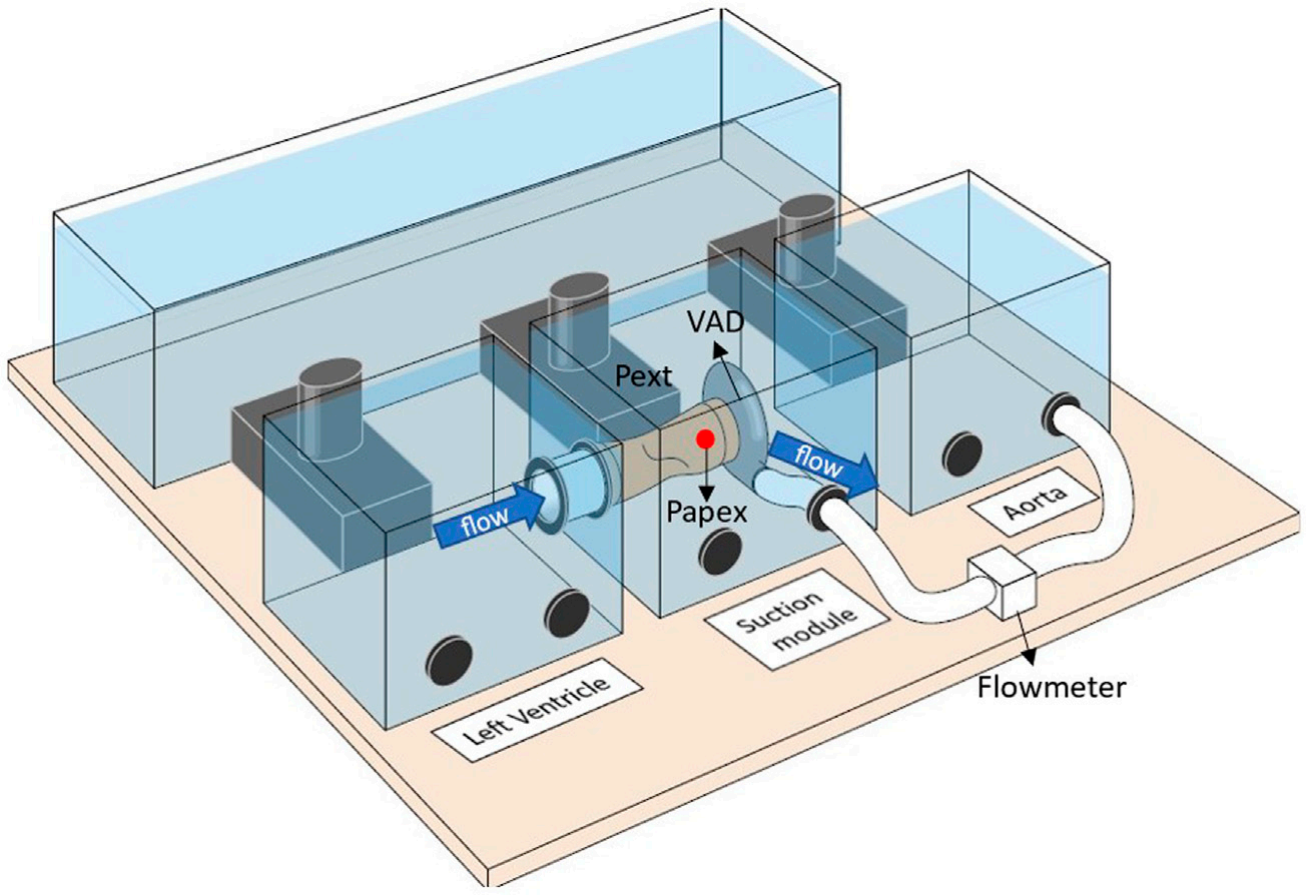
Setup of the hybrid simulator with the suction module implemented. One chamber is used to represent the flows and pressures of the left ventricle. The suction module is immersed in a chamber where it is connected between the left ventricular chamber and a LVAD system. The LVAD then ejects into a third chamber where the aortic hemodynamic is represented. A flowmeter is used to measure the LVAD flow. The pressure at the inlet cannula (Papex) is measured through a needle positioned in correspondence of the red dot. Finally, the external pressure (Pext) of the suction module is used to squeeze the tube and recreate suction when certain conditions are met during the heart cycle.

The work was conducted using the suction module (Rocchi et al., 2021a; Rocchi et al., 2021b) connected to a previously validated high fidelity cardiovascular hybrid simulator. (Fresiello et al., 2014; Fresiello et al., 2022). The hybrid simulator combines an *in silico* model to an *in vitro* system connected to each other in real time under LabVIEW environment (LabVIEW 2019 SP1, National Instruments, Austin, TX). The computational part is a lumped parameters model representing the entire cardiovascular systems (i.e., atria, ventricles, and pulmonary and systemic circulations), where both cardiac and vascular parameters can be tuned individually. The *in vitro* system is characterized by active hydraulic chambers that can reproduce the hemodynamics of any cardiovascular sites and that enable the connection of different medical devices. For this application, two chambers were used to simulate the flows and pressures of respectively the left ventricle and the aorta, whilst another chamber hosted the suction module as shown in Figure 1. The suction module is a compliant latex tube (Civco, Kalona) used to mimic the ventricular apex with a simplified geometry. It is immersed in one of the chambers of the hybrid simulator. One side of the suction module is connected to the chamber where the left ventricle is simulated, whereas the other side is connected to an HVAD system (Medtronic Inc., Minneapolis, MN, USA). The chamber containing the suction module is airtight and enables to reproduce controlled pressure profiles (Pext) acting outside the tube so to squeeze it, thus simulating suction, when the left ventricular pressure drops below 0 mmHg. (Rocchi et al., 2021a). This is the hemodynamic condition for suction to be triggered in case of ideal positioning of the inflow cannula as previously investigated *in vivo* by Salamonsen et al. (Salamonsen et al., 2015) Additional technical specifications of the suction module and the hybrid simulator were described previously. (Fresiello et al., 2014; Rocchi et al., 2021a; Rocchi et al., 2021b).

All experiments were conducted using a solution of glycerol and water (40/60% in weight) at a controlled room temperature of 21°C to achieve a viscosity and density similar to that of blood at 37°C. (Segur and Oderstar, 1951).

### 2.2 Simulations

Tests were conducted simulating two heart failure profiles: an LVAD patient with severe dilated cardiomyopathy (DCM) and an LVAD patient with restrictive cardiomyopathy (RCM). The hemodynamic profiles at baseline condition, with an LVAD speed of 2,800 rpm, are reported in Table 1:

**TABLE 1:** Hemodynamic profiles at baseline of a Dilated Cardiomyopathy and a Restrictive Cardiomyopathy. Both profiles are supported by an HVAD running at 2,800 rpm. HR = Heart rate; CO = Cardiac Output; V30 = left ventricular volume at which the left ventricular end diastolic pressure equals 30 mmHg.

		Dilated cardiomyopathy (DCM)	Restrictive cardiomyopathy (RCM)
HR	[bpm]	78	78
CO	[L/min]	4.7	5.0
Systemic Arterial Pressure	[mmHg]	87/81 (84)	102/83 (92)
Pulmonary Arterial Pressure	[mmHg]	37/16 (24)	37/10 (20)
Wedge Capillary Pressure	[mmHg]	12	7
Left Ventricular End-Diastolic and End-Systolic volumes	[mL]	183	67
	[mL]	154	13
Left ventricular End-Systolic Elastance	[mmHg/mL]	0.4	3.3
V30	[mL]	210	95

Starting from the baseline profiles, suction was induced by simulating hypovolemia. The total circulating blood volume in the cardiovascular model was reduced until suction was observed and pump flow peaks lower than 2 L/min were reached. The tests were conducted with LVAD speeds ranging from 2,500 rpm to 3,200 rpm for both DCM and RCM profiles. A total of 183 beats with suction and 19 beats without suction were recorded for the DCM, and a total of 168 beats with suction and 21 beats without suction were recorded for the RCM. Tests were done with the HVAD’s predefined periodic speed modulation (Lavare Cycle) deactivated.

For each experiment, LVAD data were recorded at a sampling frequency of 50 Hz, and hemodynamic data from the cardiovascular simulator and the suction module were recorded at 1,000 Hz using the same data acquisition systems. Hemodynamic data refer to left ventricular pressure and volume, aortic pressure, total blood volume, and pressure at the inlet cannula (Papex) as shown in Figure 1. LVAD data refer to LVAD impeller speed, motor current, power uptake, and estimated pump flow.

### 2.3 Clinical data collection

For the validation of the suction module, a database of LVAD waveforms recorded in HVAD patients enrolled in a clinical observational study at the Medical University of Vienna was used. In particular, 2,203 beats with suction and 3,931 beats without suction were recorded and included in this study. Data included were approved by the Institutional Review Board of the Medical University Vienna (EK-243/2011, ClinicalTrials.gov identifier: NCT01981642). (Gross et al., 2020; Maw et al., 2021; Moscato et al., 2021) A portable data recorder, developed at the Medical University of Vienna, was used to continuously retrieve and store the data stream for the HVAD serial monitor port. (Gross et al., 2020). Continuous LVAD data were sampled at a frequency of 50 Hz and included the impeller speed, motor current, power uptake and estimated pump flow. (Granegger et al., 2012).

### 2.4 Validation

Currently, there is not a well-defined gold standard method to identify suction events. As such, the validation process consisted of a combination of both a qualitative and a quantitative comparison between clinical and simulated data to overcome the limitations and bias of the different methods. (Walsh, 2018).

As for the qualitative analysis, simulated and clinical estimated LVAD flow waveforms were compared visually by LVAD experts (clinicians and engineers) to assess the ability of the suction module to simulate distinctive waveform characteristics of suction events both in diastole and systole.

**FIGURE 2:**
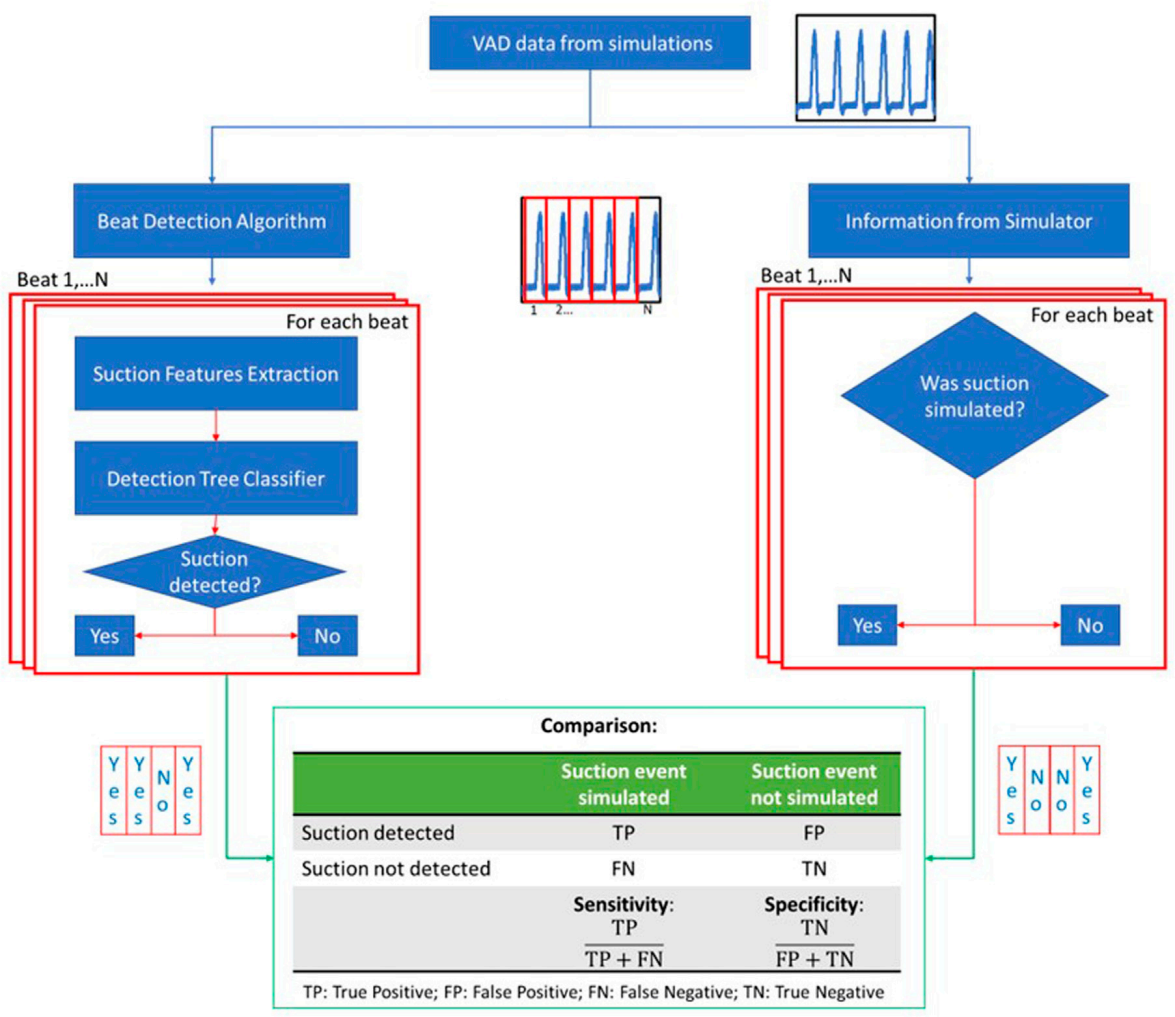
Flow chart of the quantitative analysis part 1. The waveforms of the LVAD data are fed into the beat detection algorithm where the signals are divided into beats. For each beat, suction features are extracted and used into the detection tree classifier to classify the beat either as suction or non-suction. On the other hand, the information whether suction was triggered is taken from the simulator for every beat. The two classifications are then compared to calculate specificity and sensitivity of the simulator.

The quantitative analysis consisted of two steps. In the first step (showed in the flow chart in Figure 2), the LVAD estimated flow, recorded during the simulation, was analyzed using dedicated algorithms already validated on clinical data. (Gross et al., 2020; Moscato et al., 2021). The first algorithm is the beat detector, used to segment cardiac cycles from the estimated LVAD flow signal. (Moscato et al., 2014). The second algorithm is the binary decision tree classifier, used to label each beat as suction or non-suction. This decision tree is a 3-split classifier that uses three suction features, comparing them to clinically determined thresholds. These suction features are: the minimum impeller speed pulsatility (Δω_min_), the minimum negative slope of the pump flow (dQ/dt_min_), and the timing of the maximum positive slope of the pump flow (t_dQ/dtmax_) in respect to the cardiac cycle. (Maw et al., 2021). The classifier was previously validated on clinical data using the experts’ judgment as ground truth. (Gross et al., 2020; Maw et al., 2021; Moscato et al., 2021).

In this study, the classification of simulated beats obtained from the tree classifier was compared with the simulation condition: was suction triggered or not in the considered cardiac cycle in the simulator? Specificity (true positive rate) and sensitivity (true negative rate) for the classification of the simulated data into suction beats and non-suction beats were calculated.

As a second part of the analysis, the suction features extracted from the clinical and simulated waveforms, and used in the aforementioned binary decision tree classifier, were statistically analyzed and compared. (Chase et al., 2018). First, the normality distribution for both the simulated and clinical features was checked with a Shapiro-Wilk test. Depending on the result of the Shapiro-Wilk test, either a two-tailed t-test or a Mann-Whitney U test was performed to compare clinical and simulated data.

## 3 Results

### 3.1 Qualitative analysis

**FIGURE 3:**
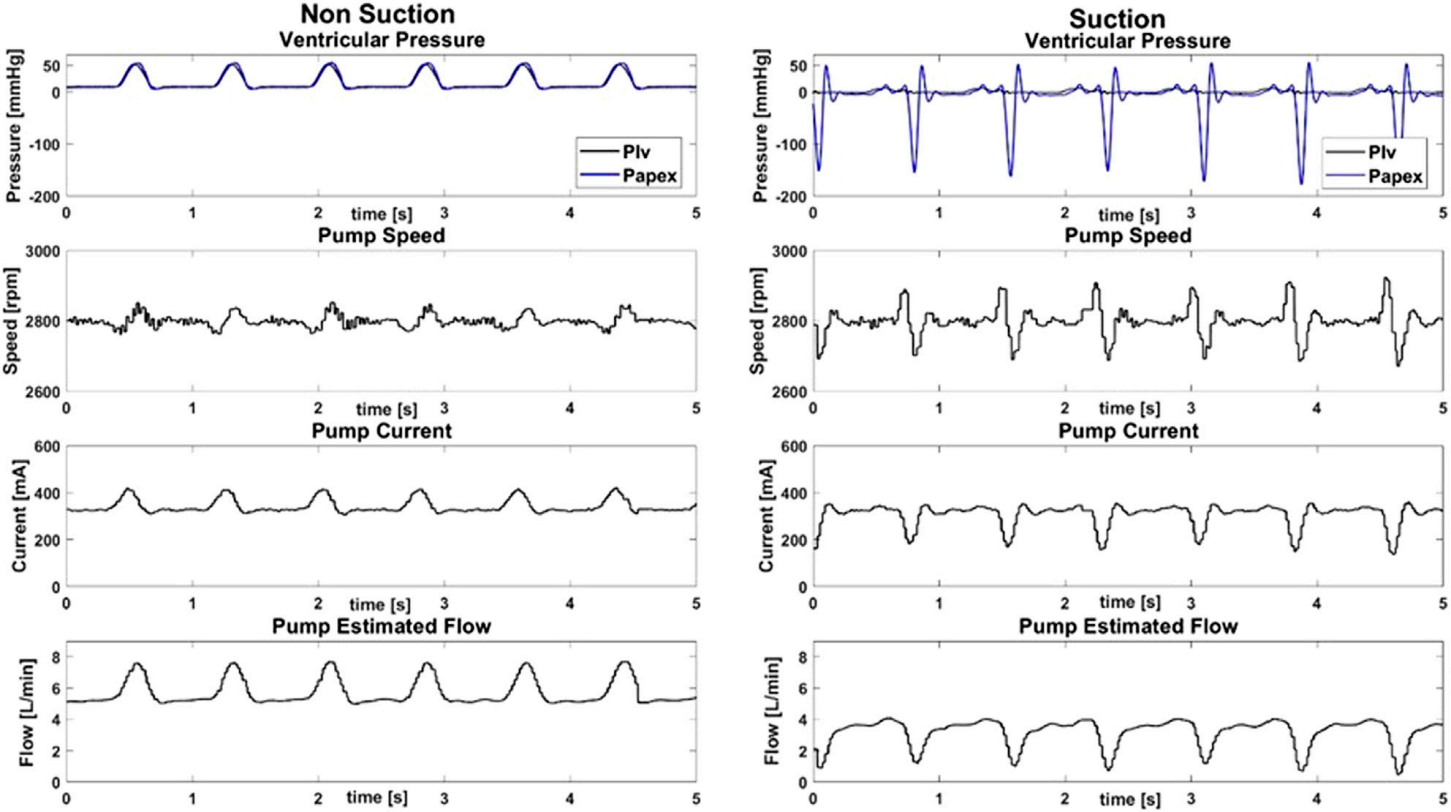
Hemodynamic data and HVAD pump data with and without suction for a simulated dilated cardiomyopathy with an HVAD speed of 2,800 rpm. In the first row, the pressure of the left ventricle (Plv, black) and of the apex (Papex, blue) are shown. The corresponding pump data: namely, the speed, the current and the estimated pump flow are shown in the second, third and fourth rows, respectively.

In Figure 3 the hemodynamic data and the LVAD data obtained simulating the DCM profile with and without suction are shown for an LVAD speed of 2,800 rpm as an example. Noticeable changes can be observed between non-suction and suction for all the reported parameters. The left ventricular pressure (Plv) and the pressure at the ventricular apex (Papex) follow each other when no suction event is identified. Conversely, during suction events, Papex drifts apart from Plv, showing a negative peak at the end of systole. During systole, with the increase in Plv, a biphasic change in speed of about ±50 rpm is noticeable when no suction occurs. In particular, a decrease in LVAD speed occurs at the beginning of systole followed by an increase in the speed at the end of systole. A different perturbation in speed is detected when suction events are present. The ventricle, in a volume deprived state, develops a smaller Plv peak during systole, according to the Frank-Starling mechanism. As such, no decrease in LVAD speed is recorded at the beginning of systole. However, in correspondence of the negative peak of the Papex, an increase in speed of ±100 rpm is observed. The trends of both the current and the estimated LVAD flow show a correlation with the Plv when no suction event is identified and are characterized by a positive peak during systole. Different trends are seen when suction events are identified. In particular, both the current and the estimated flow show a negative peak at the end of systole and lose their symmetrical shape.

**FIGURE 4:**
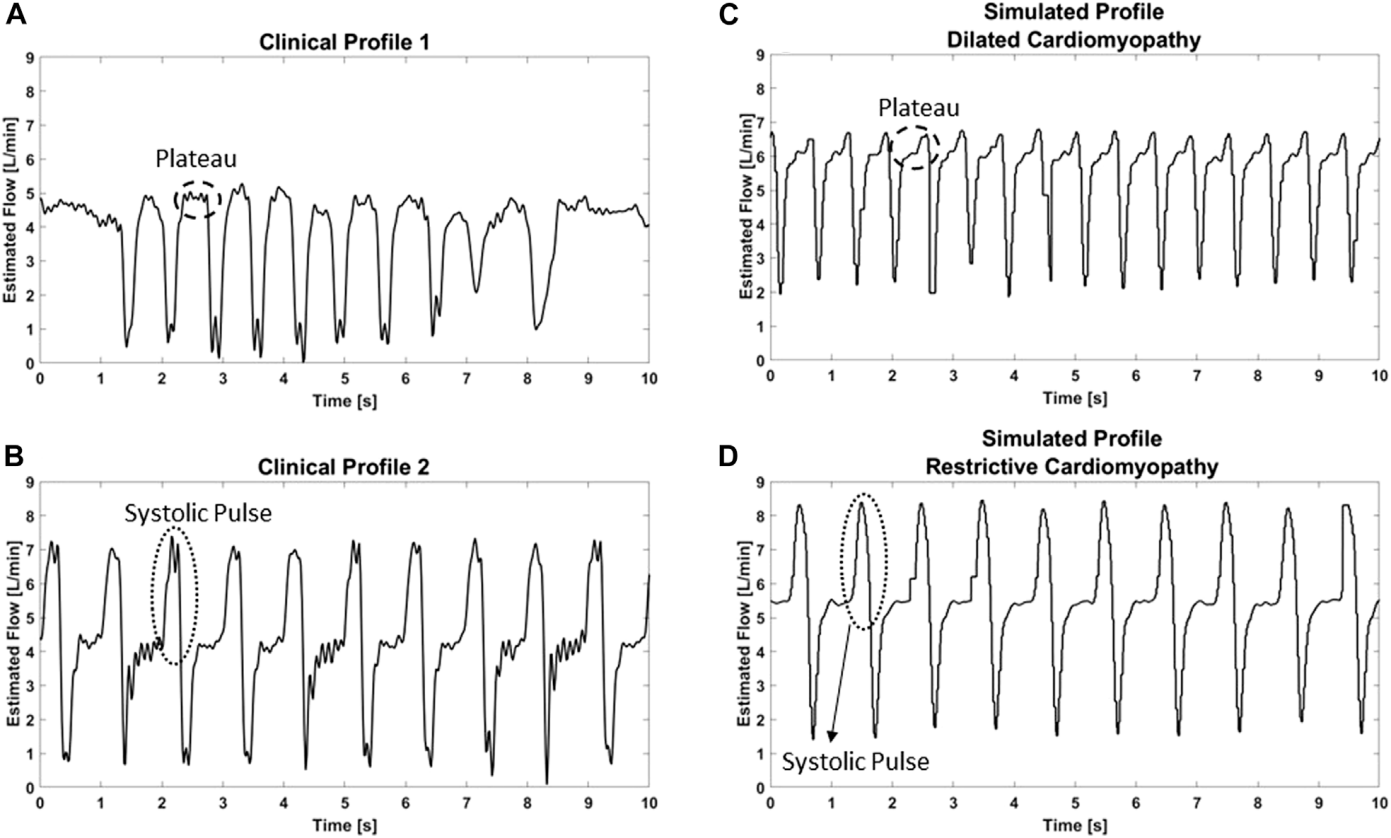
Representative clinical **(A** and **B)** and simulated **(C** and **D)** patterns of the estimated LVAD flow during suction events.

The suction module can recreate different LVAD flow waveforms seen in patients. In Figure 4 two different profiles found in clinics are shown as an example and compared to those obtained from simulations. The flow waveform obtained from the DCM simulations is comparable to the clinical profile 1 (Figures 4A,C). The waveforms are characterized by an LVAD flow transient spike at the end of systole, followed by a plateau throughout the rest of the cardiac cycle due to the poor residual contraction of the ventricle. On the other hand, the higher contractility of the left ventricle simulated in the RCM profile, develops a systolic pulse flow above the average flow level comparable to the clinical profile 2 (Figures 4B,D).

### 3.2 Quantitative analysis

The results for the quantitative analysis are discussed in the next 2 sections. The first section analyzes the results obtained with the binary classifier, whereas the second section focuses on the statistical study conducted to compare the suction features for simulated and clinical data.

#### 3.2.1 Suction detection tree classifier

The data from the simulations with different LVAD speeds, total blood volume and patient profiles were analyzed with a previously validated suction detection tree to classify suction and non-suction beats. The classifier detected a total of 316 out of 351 beats with suction and 39 out of 40 beats without suction. A good agreement between the classification given by the clinical suction detection tree and the simulator was found. In particular, the measured sensitivity and specificity of the LVAD pump data generated with the simulator resulted to be of 90.0% and 97.5% respectively.

#### 3.2.2 Suction features

**FIGURE 5:**
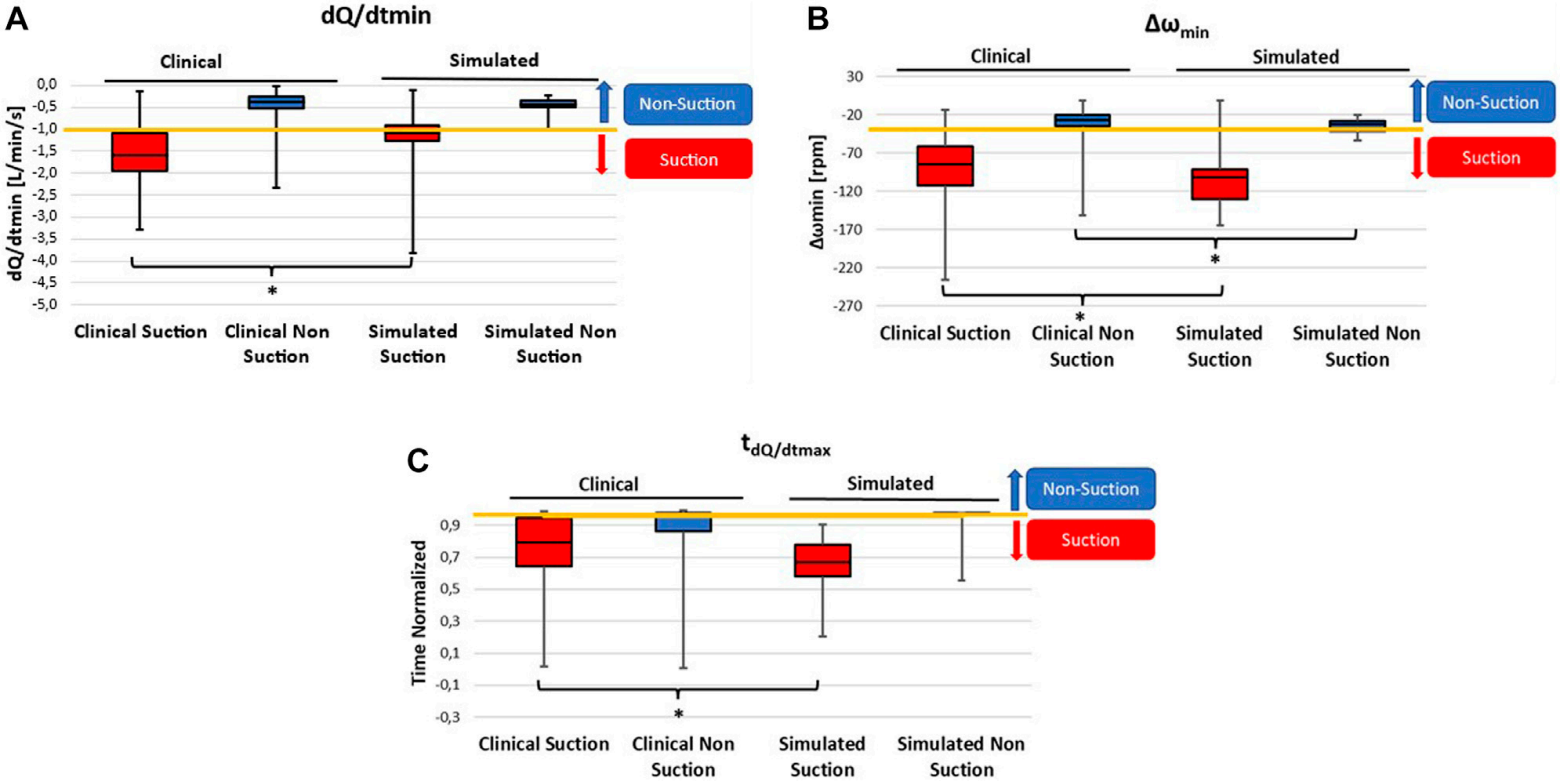
Boxplots showing the range of suction features in simulated and clinical suction (red) and non-suction (blue) beats. Data are reported as median, 25th and 75th percentiles, maximum and minimum. The features shown are the ones used in the suction detection tree: minimum negative slope of the pump flow (dQ/dtmin) **(A)**, minimum impeller speed pulsatility (Δωmin) **(B)**, and the timing of the maximum positive slope of the pump flow normalized to the beginning of diastole (t_dQ/dtmax_) **(C)**. The yellow line represents the threshold used in the binary tree classifier to distinguish suction and non-suction beats. The * symbol indicates statistical significance (*p* < 0.05) across the groups.

The suction features were extracted from the simulated beats of all experiments and from the clinical data. The Shapiro-Wilk test indicated a non-normal distribution of the data. Data are reported in Figure 5 and in Table 2 as median, 25^th^ and 75^th^ percentiles, maximum and minimum values.

**TABLE 2:** Dispersion of suction features for simulated and clinical waveforms and results of the statistical analysis to compare simulated and clinical suction features (p). Namely, the minimum negative slope of the pump flow (dQ/dtmin), the minimum impeller speed pulsatility (Δωmin), and the timing of the maximum positive slope of the pump flow normalized to the beginning of diastole (t_dQ/dtmax_). For each feature, the median, 25^th^ percentile (Q1), 75^th^ percentile, maximum (max) and minimum (min) are reported in case of suction and non-suction.

	Non-suction		Suction	
	Clinical	Simulated	Clinical	Simulated
dQ/dt_min_ [L/min/s]				
Median	−1.6	−1.1	−0.4	−0.4
Q1	−2.0	−1.3	−0.5	−0.5
Q3	−1.1	−0.9	−0.2	−0.3
Max	−0.1	−0.1	0.0	−0.2
Min	−3.3	−3.8	−2.3	−1.0
p	0.07		<0.001	
Δω_min_ [rpm]				
Median	−85	−102	−28	−33
Q1	−113	−130	−35	−42
Q3	−62	−91	−21	−28
Max	−14	−1	−2	−21
Min	−236	−165	−152	−54
p	<0.001		<0.001	
t_dQ/dtmax_ *[Time normalized]*				
Median	0.79	0.67	0.97	0.97
Q1	0.64	0.58	0.86	0.97
Q3	0.95	0.78	0.98	0.97
Max	0.99	0.90	0.99	0.98
Min	0.02	0.20	0.01	0.55
p	0.06		<0.001	

Simulated data are in the range of the clinical ones (Table 2), although clinical data characterized by a wider range compared to the simulated ones both for the suction and non-suction beats (Figure 5). Nevertheless, the median of simulated values never exceeds the values of 25^th^ and 75^th^ percentile of clinical data, both for suction and non-suction data.

In Figure 5A the ranges of clinical and simulated dQ/dt_min_ are shown. The median of dQ/dt_min_ of the simulated beats in case of suction and non-suction are respectively the 75^th^ and 38^th^ percentile of the clinical data. Simulated non-suction beats shows higher dQ/dt_min_ values with respect to the simulated suction beats, in agreement with the clinical trends.

In Figure 5B the comparison between clinical and simulated Δω_min_ is shown. Also in this case, simulated data result to be in good agreement with clinical data, with non-suction beats showing a higher median compared to the suction beats. The median of Δω_min_ of the simulated beats in case of suction and non-suction are respectively the 35^th^ and 32^nd^ percentile of the clinical data. The Δω_min_ for simulated non-suction beats is higher compared to the suction ones, as seen in the clinical data.

Finally, in Figure 5C the ranges of clinical and simulated t_dQ/dtmax_ are shown. The range covered by the simulated data is in good agreement with the range of the clinical data. The median of t_dQ/dtmax_ of the simulated beats in case of suction and non-suction are respectively the 28^th^ and 62^nd^ percentile of the clinical data.

Overall, simulated beats could be well classified by the binary decision tree. In fact, the clinically determined thresholds of the classifier for the 3 suction features can well separate the ranges of simulated suction beats from non-suction beats (Figure 5).

Given the non-normality of the data, the Mann-Whitney U-test was used to statistically compare the simulated and clinical data. The comparison does not show a statistical significance for dQ/dt_min_ and the t_dQ/dtmax_, thus demonstrating the good agreement between clinical and simulated data when no suction is identified (see Table 2; Figure 5). However, the comparison of all the features during suction showed a statistically significant difference.

## 4 Discussions

The aim of this study was to validate the suction module connected to a hybrid cardiovascular simulator present at KU Leuven. (Rocchi et al., 2021a; Rocchi et al., 2021b). For this purpose, suction beats and non-suction beats were simulated with a HVAD for different pathophysiological conditions. The results of these experiments were compared against clinical data of 38 HVAD patients in suction and non-suction conditions. (Gross et al., 2020; Maw et al., 2021; Moscato et al., 2021).

Due to the lack of a gold standard validation method (Walsh, 2018), the comparison between simulated and clinical data was conducted at various levels: first the time trends of the LVAD estimated flow waveform were qualitatively compared, and then specific features of the pump data were quantitively analyzed.

The pump waveforms obtained from the simulations have similar trends compared to those recorded in the clinics in both suction and non-suction conditions, as indicated in Figure 4. (Karantonis et al., 2007; Hayward et al., 2015). In particular, when suction does not occur, the estimated LVAD flow increases during the systolic contraction. The pump speed decreases initially, as a result of a change of torque on the impeller, followed by a sharp rise in early systole due to the action of the motor controller to maintain a constant pump speed. In case of suction events, different trends are identified. In particular, suction events cause inflow occlusion leading to an initial drop of the pump flow and a subsequent increment in the pump speed. Then, a rapid decrease in the speed is noticed as a consequence of the action of the motor controller to restore the LVAD speed to the setpoint value.

These changes in LVAD signals have been reported as significant to detect suction events. Some research groups and companies developed algorithms using features extracted either from a single pump parameter such as the motor current, the pump speed or the estimated flow (Hatoh et al., 1999; Vollkron et al., 2004; Karantonis et al., 2006; Ferreira et al., 2007) or a combination of the previous. (Maw et al., 2021).

In this study, the 3-split decision tree developed by Gross et al. (Gross et al., 2020) was used to classify suction and non-suction beats. A total of three features (Δω_min_, the dQ/dt_min_, and the t_dQ/dtmax_) are used in the decision tree, which shows an accuracy of 97.7% in detecting suction and non-suction beats. (Gross et al., 2020; Maw et al., 2021; Moscato et al., 2021).

Moreover, we analyzed these same features as part of the simulator validation process, and assessed to what extend the Δω_min_, the dQ/dt_min_, and the t_dQ/dtmax_ from simulated beats resembled the values from clinical beats. These features extracted from the simulator were statistically compared to the ones found in patients.

From the data obtained in the simulations, the Δω_min_, the dQ/dt_min_, and the t_dQ/dtmax_ features are all in the interquartile range of their corresponding clinical data, for both suction and non-suction beats. As a result, the decisional tree can well distinguish between suction and non-suction simulated beats. In particular, the majority of the suction beats are classified in the first branch of the 3-split classifier. Namely, 85.7% of simulated suction beats are classified as suction by the dQ/dt_min_. This result is comparable to the one obtained for the classification of clinical data, where 91.3% of data were classified as suction in the first branch of the detection tree classifier. The classification performance on waveforms generated with the simulator is matched with a 90.0% sensitivity and 97.5% specificity.

Although simulations produced LVAD features in the range of clinical ones (within 25^th^ and 75^th^ percentiles), the Mann-Whitney U test shows a different distribution between simulated and clinical data for most of the suction features. Clinical data show a wider variability for the considered features, which can only be partly captured in the simulation domain. This variability could have various sources, from the LVAD positioning in the ventricular chamber to the patient’s hemodynamic status, and it is challenging to clinically identify them, as LVAD recordings are usually conducted in a daily life setting, without any additional clinical measurement.

To tackle this variability, we focused our investigation on the ventricular status and simulated the two antipodes of the heart failure spectrum: an RCM with small ventricular chamber and high contractility and a DCM with large ventricular chamber and low contractility. Interestingly, these two profiles produced quite different suction phenomena, in line with those observed in the clinics (Figure 4). For a DCM profile, suction exhibits a significant low LVAD flow peak at the end of systole followed by a plateau throughout the remaining cardiac cycle. A different LVAD flow pattern is observed in case of the RCM profile, with a noticeable systolic pulse resulting from the ventricular contraction.

According to our results, left ventricular systolic and diastolic functions do play a great role in determining the phenomenology of suction, but we do not exclude the influence that other factors such as right heart failure, tachycardia, or hypotension might have on the LVAD signals. (Rocchi et al., 2021b). This could be a reason why our simulated cohort, focused on ventricular parameters only, represents just a subgroup of the LVAD signals variability observed in the clinic. Another reason for the wider clinical variability of LVAD signals could be the (mal)positioning of the inflow cannula in the ventricle. Suction events caused by cannula malpositioning generally show larger pulsatility, and they occur during near normal left ventricular filling and preload. (Gross et al., 2020). In our simulator this phenomenon is not captured, and the cannula is assumed to be in the ideal position so that suction occurs only when the ventricle empties completely.

It goes without saying that capturing the entire variability of the suction phenomenology is important, as it is relevant to understand why this variability is present, and to analyze its underlying mechanisms. The variability of suction phenomenology should be considered by companies and research groups when developing algorithms to detect and mitigate suction. (Hatoh et al., 1999; Vollkron et al., 2004; Karantonis et al., 2006; Ferreira et al., 2007; Maw et al., 2021; Maw et al., 2022). This study has shed the light on how different ventricular properties (DCM and RCM) can induce very distinct suction patterns in case of hypovolemia. Other studies should follow to investigate other clinical conditions during suction, and possibly identify specific suction features for each of them. This knowledge could lead to the development of an algorithm not only to detect suction, but also to identify its underlying cause, and give information on how to best treat the patient accordingly. (Rocchi et al., 2021b).

Besides patient related hemodynamic and anatomical factors, suction features are impacted also by LVAD related factors such as the design and material of the inflow cannula, the pump pressure-flow characteristics, the pump control operation, and the presence of speed modulation algorithms (i.e., Lavare Cycle). All these factors might alter the magnitude of suction and its dynamic features. (Gregory et al., 2011; Ferreira et al., 2012; May-Newman et al., 2019). Even though our investigations are framed to the HVAD only, we might infer that our findings are extendable also to other systems based on rotary blood pump technology. All pumps with this technology have the inherent limitation of low preload sensitivity, therefore suction is based on the same phenomenological events observed for the HVAD. On this regard, the suction module can be adapted to any LVAD (by interchanging the connector to hold the pump in the hydraulic chamber) so to investigate differences among LVAD models, given a same and repeatable set of patient hemodynamic condition.

Indeed, simulators can offer a versatile and repeatable set up where different case scenarios and extreme hemodynamics can be recreated. Such set ups allow the comparison of different design of pumps, inflow cannulas, and/or control algorithms under the same hemodynamic conditions. As such, the use of an *in vitro* model capable of mimicking suction events, can support the developmental and testing phases of LVAD controllers before their implementation in the clinic.

## 5 Conclusion

Overall, the suction module implemented in the hybrid simulator showed a good agreement with the clinical data. The two heart failure profiles reproduced with the simulator showed the capabilities of the suction module to replicate different mechanical properties of the left ventricle. As such, the suction module can be a valuable test bench to investigate the suction phenomenon, to test physiological LVAD controllers.

## Data Availability

The original contributions presented in the study are included in the article/supplementary material, further inquiries can be directed to the corresponding author.
